# Contraceptive access in displacement settings: a quantitative study of Syrians displaced to Türkiye

**DOI:** 10.1080/26410397.2025.2607838

**Published:** 2026-01-07

**Authors:** Rosanna Le Voir

**Affiliations:** PhD Candidate, Department of Methodology, London School of Economics and Political Science, London, UK.

**Keywords:** Syrians, displacement, sexual and reproductive health, contraception, access, data

## Abstract

Access to contraception for displaced populations is both lifesaving and a right. This paper argues that displacement demands a separate analytical lens from other mobilities and crisis contexts. I offer a framework, based on established concepts and available evidence, to understand different aspects of contraceptive access in displacement. I then use the case study of Syrians displaced to Türkiye, a population for whom data quality and availability is comparably better than other displacement settings, as a worked example to test the framework using empirical analysis of nationally representative survey data. I analyse contraceptive use and reasons for non-use as a proxy for access among married women, optimising data from the Syrian sample of the 2018 Türkiye Demographic and Health Survey (*n* = 1736) and the 2006 Syria Multiple Indicator Cluster Survey (*n* = 13,619). The results show that the most relevant dimensions of the framework that constrained access to displaced women’s preferred methods of contraception were cognitive accessibility and perceived quality of care, specifically fear of side effects and other health concerns. A minority of women who were currently using contraception still faced barriers in accessing their preferred method, suggesting limits to contraceptive autonomy. This case study offers theoretically transferable findings about data and evidence for other displacement settings. Notably, the present data landscape on the sexual and reproductive health of displaced populations does not adequately capture issues around contraceptive access and understandings will remain partial with the currently available metrics.

## Plain language summary

The number of people forced to leave their homes due to conflict and violence is higher than ever. In displacement, people continue to have everyday needs. These include information and services about contraception, should they wish to use it. Access to these services can vary depending on the context and duration of displacement. This paper offers a framework for contraceptive access in displacement. The framework outlines factors that may help or restrict access, such as cost and awareness of services. I use the example of Syrians displaced to Türkiye, following the escalation of the Syrian conflict in 2011, to analyse different types of access. In this displacement context, fear of side effects and other health concerns can be a barrier to using contraceptives. Some Syrian women in Türkiye who were using contraception could not access their preferred method. This paper focuses on Syrians in Türkiye, but the framework can be applied to other displacement settings. Currently, the availability of information is a barrier to understanding contraceptive access. I provide recommendations for how to strengthen the data.

## Résumé

L’accès à la contraception est vital pour les populations déplacées et c’est aussi un droit. Cet article avance que le déplacement exige une perspective analytique différente de celle utilisée pour d’autres formes de mobilité et contextes de crise. Je propose un cadre de travail, fondé sur des concepts établis et les données disponibles, pour comprendre les différents aspects de l’accès à la contraception en situation de déplacement. J’utilise ensuite l’étude de cas de Syriens déplacés en Türkiye, population pour laquelle la qualité et la disponibilité des données sont comparativement meilleures que pour d’autres contextes de déplacement, comme exemple pour tester ce cadre au moyen d’une analyse empirique de données d’enquêtes représentatives au plan national et accessibles au public. J’analyse l’utilisation de la contraception et les raisons de la non-utilisation comme variable d’accès chez les femmes mariées, en optimisant les données de l’échantillon syrien de l’Enquête démographique et de santé de la Türkiye de 2018 (*n* = 1736) et l'Enquête par grappes à indicateurs multiples de la Syrie de 2006 (*n* = 13,619). Les résultats montrent que les dimensions les plus pertinentes du cadre qui limitaient l’accès des femmes déplacées à leurs méthodes de contraception préférées étaient l’accessibilité cognitive et la qualité perçue des soins, plus précisément la crainte d’effets secondaires et d’autres préoccupations de santé. Une minorité de femmes utilisant alors une contraception continuaient à rencontrer des obstacles pour accéder à leur méthode préférée, ce qui suggère des limites à leur autonomie en matière de contraception. Cette étude de cas offre des conclusions théoriquement transposables en matière de données et d’indicateurs dans d’autres contextes de déplacement. Il convient de noter que le tableau actuel de données sur la santé sexuelle et reproductive des populations déplacées ne rend pas suffisamment compte des problèmes d’accès à la contraception et que les connaissances resteront partielles avec les indicateurs actuellement disponibles.

## Resumen

El acceso a métodos anticonceptivos para poblaciones desplazadas no solo salva vidas, sino que es un derecho. Este artículo argumenta que el desplazamiento exige una perspectiva analítica diferente a la de otros contextos de movilidades y crisis. Ofrezco un marco, basado en conceptos establecidos y en la evidencia disponible, a fin de entender los diferentes aspectos del acceso a métodos anticonceptivos para personas desplazadas. Utilizo el estudio de caso de sirios desplazados a Türkiye, una población para la que la calidad y disponibilidad de datos es comparablemente mejor que en otros entornos de desplazamiento, como un ejemplo práctico para probar el marco utilizando el análisis empírico de datos de encuestas representativos a nivel nacional. Analizo el uso de anticonceptivos y las razones para no usarlos como un proxy para el acceso entre mujeres casadas, y optimizo los datos de la muestra siria de la Encuesta Demográfica y de Salud de 2018 de Türkiye (*n* = 1736) y la Encuesta de Indicadores Múltiples por Conglomerados de 2006 de Siria (*n* = 13,619). Los resultados muestran que las dimensiones más pertinentes del marco que limitaron el acceso de las mujeres desplazadas a sus métodos anticonceptivos preferidos eran accesibilidad cognitiva y calidad percibida de la atención, en específico miedo a tener efectos secundarios y otras preocupaciones de salud. Una minoría de las mujeres que actualmente utilizaban anticoncepción aún enfrentaban barreras para acceder a su método preferido, lo cual indica que hay límites a la autonomía anticonceptiva. Este estudio de caso ofrece hallazgos transferibles teóricamente sobre los datos y la evidencia para otros entornos de desplazamiento. Cabe señalar que el panorama actual de datos sobre la salud sexual y reproductiva de poblaciones desplazadas no captura adecuadamente los aspectos relacionados con el acceso a métodos anticonceptivos y que, con las métricas disponibles actualmente, la comprensión seguirá siendo parcial.

## Introduction

### Comprehensive contraception in displacement settings

Over the last 30 years, there has been greater recognition of the life-saving and rights-based imperative for supporting sexual and reproductive health (SRH) in conflict and displacement settings.^1^ Inadequacies in both the services for, and evidence on, the SRH of populations living in these settings are well established.^2–5^ This increased attention is reinforced by international standards and guidance such as the Inter-Agency Field Manual on Reproductive Health in Humanitarian Settings,^6^ Minimum Initial Service Package for SRH in Crisis Situations,^7^ and the Granada Consensus on SRH during protracted crises and recovery.^8^

Living in conflict and fragile settings can have a detrimental impact on SRH, including access to safe, quality, and affordable contraceptive information and services.^9^ Different dimensions of SRH may be affected unevenly; case study evidence from 10 conflict-affected countries found deprioritisation of reproductive health services, including contraception, relative to other maternal and child health services.^10^ Ensuring people can access contraception, should they wish to use it, helps to reduce unintended pregnancies, unsafe abortion, and maternal deaths.^11^ This is especially relevant in conflict settings where mortality among women of reproductive age is significantly higher than in peaceful settings.^12^

A comprehensive approach to contraceptive information and services emphasises the centrality of informed choice and reproductive autonomy.^13^ This includes – but is not limited to – counselling on the range of contraceptive methods available and possible side effects, as well as the freedom to discontinue, switch, or not use a method.^14,15^ A comprehensive approach considers both short- and long-acting modern methods, emergency contraception, and so-called traditional methods that use fertility awareness. An absence of this comprehensive approach can reinforce risks of coercion, including psychological pressure to prevent or promote pregnancies, as well as other forms of reproductive violence.^16,17^ This is especially relevant to displaced people, a population whose everyday practices show extraordinary resilience and agency,^18^ but who may be subject to unequal power relations and disempowered through their precarious legal (and other) status. Comprehensive care can help to ensure that their rights are met and protected, rather than being a group whose fertility is controlled.^19,20^ In a recent research prioritisation exercise, the World Health Organization (WHO) identified “comprehensive contraceptive services” in humanitarian settings as a global research priority.^21^

### Distinguishing displacement from other mobilities and crisis contexts

Existing literature often refers generally to humanitarian, fragile and/or conflict-affected contexts. These settings are increasingly complex and hard to define, spanning a wide range of rapid onset emergencies, protracted crises, localised disasters, and regional conflicts. Population displacement is a common feature of these contexts, with more than 120 million people estimated to be forcibly displaced at the end of 2024.^22^ Meanwhile, the United Nations (UN) estimates that a much larger group, more than 300 million people globally, needed humanitarian assistance and protection in 2025.^23^ I argue here that displacement demands a separate lens, rather than being studied together with a myriad of other contexts and mobilities, for three main reasons.

First, while acknowledging that the complexity of human mobilities does not fit neatly into definitions and categories,^24^ it can be helpful to distinguish displacement from other migratory movements. People who move due to drivers involving some element of force, compulsion, or coercion^25^ may find themselves in contexts very different to those who move for reasons mainly due to education or employment. People can be displaced for different or multiple reasons. This paper focuses on conflict and violence, the main drivers of displacement globally. However, displacement can also occur due to environmental degradation and climate change; natural disasters; socio-economic and state fragility, as well as development and construction.^26^

Second, displaced people themselves are highly heterogeneous, and definitions and categories of displacement are complex and political.^26^ Displaced persons include those labelled as refugees, with legal protection under the 1951 Convention Relating to the Status of Refugees and 1967 Protocol, as well as internally displaced persons (IDPs), asylum seekers, stateless people, and those with precarious status. These categories partly relate to whether people are displaced within their country (i.e. IDPs) or move across international borders. But not all displaced people share the same legal status. For example, displaced Syrians in Türkiye are given “temporary protection status” rather than recognition as refugees.^27^ Similarly, the category of “Venezuelans displaced abroad” are not officially counted as asylum seekers, refugees, or “others of concern” by the UN Refugee Agency (UNHCR).^28^ A specific focus on displacement provides more space to recognise this heterogeneity.

Finally, wider debates across the international community have challenged the traditional humanitarian framing of displacement. In the past, siloed approaches have led to separate humanitarian data sources, coordination mechanisms and response plans, often operating outside of national systems.^29^ Recognising the protracted nature of displacement, with most displacement lasting more than five years, the UN and international community have advocated for longer-term, locally-owned solutions across the humanitarian-development “nexus”.^30–32^ This includes calls to reduce the methodological divide between humanitarian and development data, and strengthen alignment with international statistical standards.^33^ By studying displacement outside of a humanitarian-focused lens, I acknowledge how it transcends artificial humanitarian-development siloes.

### Gaps in the literature and study objectives

Existing studies that focus on refugees and IDPs are often localised quantitative studies at a specific site (e.g. hospital or camp) or in-depth qualitative studies. The gap in larger scale, representative quantitative evidence can be linked to the technical, ethical, and operational barriers to the measurement of displacement (e.g. absence of sampling frames), as well as siloes in humanitarian-development data systems, contributing to poor data availability.^29,34,35^ Efforts have been made to optimise existing, albeit imperfect, data to better understand SRH outcomes of displaced people, as well as highlight the importance of displacement as an analytical lens.^36^ A nationally representative study in Iraq found that displaced women who previously lived in a camp were almost half as likely to use modern contraception compared to those who previously lived in a city, town or rural area.^37^ However, the mechanisms that facilitate or constrain contraceptive access among displaced people have not been thoroughly explored.

This paper addresses these gaps in the literature, advancing theoretical and substantive understandings of different aspects of contraceptive access in displacement settings. In the rest of this paper, “displacement” refers to displacement due to conflict and violence, including movement of people within and across borders. First, I offer a framework, based on established concepts and available evidence, helping to unpack general notions of contraceptive access in displacement. Second, I test components of the framework using empirical analysis of nationally representative survey data. I analyse reasons for contraceptive non-use as a proxy for different dimensions of access in the framework. I also show how patterns of contraceptive use among displaced Syrians in Türkiye differ from the most closely comparable non-displaced population. The case study of displaced Syrian women offers the opportunity to see how much we can learn about contraceptive access among a population for whom data availability and quality is comparably better than other displacement settings. The specific lens on displacement provides a more focused approach than humanitarian or fragile settings more broadly, and recognises how displacement transcends the artificial humanitarian-development dichotomy.

### Study setting: Syrians in Türkiye

Government data for November 2025 estimates that Türkiye hosts 2.4 million Syrian refugees, of whom almost three-quarters are women and children.^38^ The displacement situation is protracted, reflecting Türkiye’s open-door policy to Syrians since the outbreak of conflict in Syria in March 2011.^39^ While the displacement of Syrians is characterised as due to conflict and violence, it is important to note that the Syrian population in Türkiye is not currently in conflict; they fled from it. Türkiye does not recognise non-EU asylum seekers, including Syrians, as refugees, instead they are given “temporary protection status”.^27^ This category gives equivalent access to services to those with refugee status but is not recognised by international law. A minority of Syrians in Türkiye, around 48,000 in 2023, live in temporary accommodation centres including refugee centres, tents, or container cities.^40^

Before the outbreak of conflict in 2011, Syria had a relatively strong health system and contraceptive services were available free of charge in public facilities. A series of national surveys document a steady increase in current contraceptive use among married women (modern and traditional methods combined), from 30% in 1978, 40% in 1993, 47% in 2001, to 58% in 2006.^41–44^ A handful of articles chart the progression of SRH services for Syrians across this period.^41,45–48^ The availability of contraceptives expanded in the 1980s in pharmacies and Ministry of Health facilities, in partnership with the Syrian Family Planning Association.^45^ Together with broadcasts in support of family planning on the official radio station, this helped to legitimise the use of contraceptives, and between 1983 and 1988, the number of new users of family planning increased by seven-fold.^45^ At the 1993 Arab Population Conference in Amman, the Syrian delegation noted family planning to be a principal component of primary health care.^45^ On the eve of the crisis, Syria’s 2007–2011 UN Development Assistance Framework dedicated an explicit goal to access and use of SRH services.^49^

Literature documents the health service provision, utilisation, and outcomes for Syrians after their displacement to Türkiye.^50–56^ Syrians with temporary protection status are eligible for free health services; this was initially restricted to the province where they are registered, but there have been efforts to expand free access throughout the country.^50^ Primary, secondary, tertiary and emergency care are available at public hospitals and migrant health centres, including for those in temporary accommodation centres.^54^ SRH services include contraceptive counselling, information leaflets in Arabic, contraceptive commodities, newborn screening, as well as free iron and vitamin D supplements for pregnant and postpartum women.^27,50^ At the time of writing, it is unclear if the full range of contraceptive commodities, including emergency contraception, is freely and consistently available across all areas and health centres. Syrian doctors can work in migrant health clinics.^57^

Studies on pregnancy and obstetric outcomes at facility- or sub-national-level found notable differences for Syrian compared to Turkish women.^51^ This includes higher likelihood of low birth weight, preterm birth, adolescent pregnancy, and anaemia for Syrians.^58–64^ Barriers to health services include language, population mobility, and some administrative restrictions, for example when registration is incomplete and there is no fixed address.^27,50^ A 2020 scoping review found only one study providing representative data on the sexual and reproductive health of Syrian women in Türkiye^51^; it was published more than a decade ago, before subsequent increases in the displaced Syrian population.^65^

The 2018 Türkiye DHS (TDHS) enabled a big step forward in knowledge production on the demography of Syrians in Türkiye.^66^ This includes documenting higher overall, and adolescent, fertility among Syrian compared to Turkish women, and a positive integration effect on Syrian children’s health outcomes as they spent more years in Türkiye.^67^ To date, I have not found peer-reviewed academic or grey literature that tests frameworks of contraceptive access in displacement using empirical analysis, including among Syrians in Türkiye.

## Conceptual framework: contraceptive access in displacement

The concept of access dominates literature and policy debates in health. The mission statement of the Inter-Agency Working Group on Reproductive Health in Crises (IAWG) is “to strengthen and expand access to quality sexual and reproductive health services for people affected by crises”.^68^ The concept of access is also included in the UN Sustainable Development Goal metrics for SRH, via targets 3.7 and 5.6.^69^ In 2018, the Guttmacher–Lancet Commission on Sexual and Reproductive Health and Rights made an explicit link between rights and access, highlighting how “all individuals have a right to make decisions governing their bodies and to access services that support that right” (p. 2646).^11^

Despite this concept’s widespread use, there has been limited attention to the mechanisms that facilitate or constrain access to contraceptive services specifically in displacement settings. In this section, I first provide a brief overview of the chronology of development of the concept of access in health. I then offer a framework, based on well-established domains of access and available evidence, to interpret the specific situation of displaced people in relation to contraceptive access.

Access is a complex and contested concept, with evolving interpretations that constitute “the battle of the frameworks”.^70^ This is because access can refer to a whole range of factors that may constrain or facilitate health outcomes, intersecting in different ways at the individual, community, and structural levels. Major contributions in the literature include the “Five As” model^71^; distinctions between “access to services”, “quality of care” and “medical barriers”^72^; the rights-based availability, accessibility, acceptability and quality (AAAQ) framework^73^; and a patient-centred approach incorporating dimensions of “abilities” alongside access.^74^ Choi et al^75^ synthesised some of these established frameworks and assessed the feasibility of measuring key elements using Demographic and Health Survey (DHS) data from sub-Saharan Africa.^75^ Sochas^76^ developed Choi et al's work further, outlining seven key dimensions of geographic and social access in multidimensional health system environments: cognitive, psychosocial, geographic, and administrative accessibility; affordability; perceived quality of care; and availability.^76^

These seven dimensions of access are not intended to imply a shortcoming in individuals’ capacity or knowledge. Rather, rights-based approaches emphasise that underlying structural and systemic issues may be at play. For example, cognitive accessibility, defined as the “extent to which potential clients are aware of the locations of service (…) points and of the services available at these locations”,^72,76^ may be constrained by women’s access to education, health education, and weaknesses in the wider health system. These factors, rather than individual capacities, may result in less awareness of specific health facilities or contraceptive methods.

Early frameworks tailored to SRH outcomes in conflict and displacement make brief references to “access to services”^77^ and “access to healthcare”,^78^ but do not unpack the concept further. A recent scoping review and framework on SRH access pooled forcibly displaced populations together with other types of migrants (e.g. immigrant women working in a business).^79^ The authors highlight that this is a key limitation of the paper, as the challenges in accessing SRH services may be different for each population. Their study also excludes systematic reviews and key references such as evidence on displaced populations from the IAWG 2012–2014 global evaluation on reproductive health in crises.^80,81^

**Figure 1.**
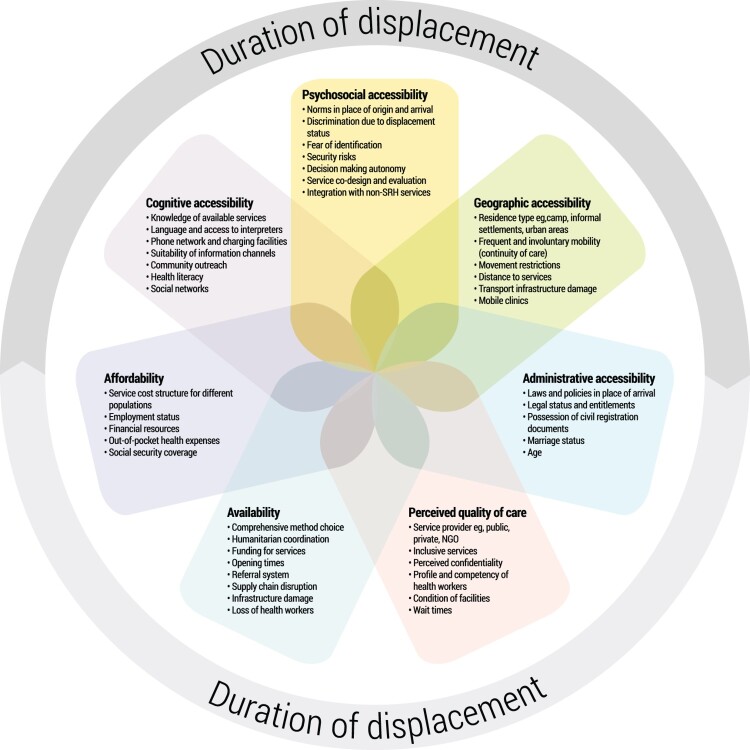
Aspects of contraceptive access in displacement settings

In Figure 1, I outline a framework for understanding different aspects of contraceptive access in displacement settings. The framework is structured around seven well-established dimensions of access that span both structural and individual factors: cognitive, psychosocial, geographic, and administrative accessibility; affordability; perceived quality of care; and availability.^76^ These seven dimensions also align with the AAAQ framework,^73^ teasing apart availability, accessibility, acceptability and quality, and some of the sub-dimensions of these. I draw on peer-reviewed evidence to interpret the specific situation of displaced people in relation to access to contraceptive services.^2–5,51,79,82–96^ The framework is intentionally flexible to be relevant across diverse geopolitical displacement settings. Appendix 1 provides detail on the methodology to develop the framework, and a table with examples from the literature.

Whilst populations living in these contexts share some similarities – for example, the majority are in low- and middle-income countries – they are also highly heterogeneous.^23^ Individual characteristics such as age, wealth, disability, gender, and sexual orientation may intersect in different ways across the seven dimensions of access outlined in Figure 1. Moreover, one person may be disadvantaged across multiple axes simultaneously. For example, unmarried adolescents may experience greater stigma in seeking contraceptive services compared to married adult women.^97^ Geographic access may be more of a barrier for displaced persons who require wheelchair accessible transport, compared to those who do not.^98^ The intersectoral nature of these individual characteristics – and how they relate to quickly changing, volatile displacement environments – are why some factors may both facilitate and constrain contraceptive access.

The duration of displacement can also vary substantially, with different dimensions of access evolving over time. IAWG refers to the continuum of an emergency, transitioning from the initial response to restore basic SRH services and moving towards comprehensive services.^97^ Time is especially relevant considering most displacement becomes chronic and protracted; Syrians displaced to Jordan, Lebanon and Türkiye have now been there for at least 10 years, while Palestinian refugees have been displaced for decades.^99^ Reduced funding over time may constrain the availability of contraceptive services, as the initial influx of resources at the onset of a crisis inevitably falls. For example, the health cluster appeals for the displacement crisis in Chad, which includes SRH, was activated in 2007 and as of December 2023, only 18% funded.^100^ Time may also enable stronger integration with existing public services, where host society “opportunity structures” allow^101^ through increased availability, geographic and administrative access. The largely protracted nature of displacement does not mean that it is a fixed situation or static process. People may experience single or multiple displacements – there is no linear “on the move” route.

In this paper so far, I have explained the value of distinguishing displacement from other mobilities and crisis contexts. I have also introduced the study setting and outlined a framework of factors that constrain and facilitate contraceptive access in displacement (see Figure 1). The next section of the paper turns to the reality of the current data landscape on the SRH of displaced populations. I use the case study of Syrians, a population for whom data quality and availability is relatively strong compared to other displacement settings, as a worked example to test the framework using empirical analysis of existing nationally representative data.

## Methods

### Data

This study uses data from the Syrian migrant sample of the 2018 TDHS and the 2006 Syrian Arab Republic Multiple Indicator Cluster Survey (MICS).^44,102,103^ DHS and MICS are both nationally representative household surveys that are coordinated internationally and implemented by national institutions with technical assistance from the DHS Program and UNICEF, respectively. MICS data referenced in the paper are available from UNICEF at https://mics.unicef.org/surveys, with free registration. TDHS data is available to researchers through a data request to the Haceteppe University Institute of Population Studies. Ethics committee approval and additional informed consent were not required for this secondary analysis of existing survey data.

The 2018 TDHS is the first national survey conducted in Türkiye to include a Syrian migrant sample. It provides data on SRH indicators among Syrian women of reproductive age 15–49 (*n* = 2216) in displacement, including those who are currently married or in union (*n* = 1736). The 2018 TDHS asks detailed individual-level questions on migration history and place of birth. A review of national surveys on SRH in displacement identified the 2018 TDHS as one of the strongest examples in the UN Asia region.^36^ This detailed unique dataset therefore offers the potential for more nuanced perspectives, compared to previous analyses of national survey data on reproductive health in displacement.^37^ The 2006 MICS was conducted before the escalation of the Syrian conflict in 2011. It offers a baseline perspective of the SRH of currently married or in union women in Syria (*n* = 13,619), including disaggregation by governorate. Together, the two datasets offer potential for analysis of a displaced population, compared to the most closely comparable non-displaced population in the country of origin.

### Variables

The 2018 TDHS includes a wider set of questions on contraception than the 2006 MICS. For both surveys, I use current contraceptive use as an outcome variable. Adopting a comprehensive lens to contraception, this includes both traditional and modern methods, and disaggregation by method type (short- and long-acting, individual methods). The 2006 MICS and 2018 TDHS use largely consistent categorisation of modern methods (female sterilisation, pill, IUD, injectables, male condom, diaphragm/foam/jelly) and traditional methods (periodic abstinence/rhythm, withdrawal) of contraception. The only differences are (1) 2006 MICS does not include emergency contraception and (2) 2018 TDHS categorises lactational amenorrhoea method (LAM) as a modern, rather than traditional method. I recode LAM to modern methods in the 2006 MICS for consistency.

The 2018 TDHS includes additional questions on reasons for not using contraception. Among current users, I use indicators about those who would prefer to use an alternative method than the one they are currently using, the preferred method, and the reason they are not using it. For those not currently using contraception, I analyse variables on reasons for contraceptive non-use and discontinuation of last method in the previous five years. Together, these variables on contraceptive non-use act as proxies to operationalise the concept of contraceptive access.^75^ I recognise that access and (non-)use are not synonymous; for example, an individual could seek and obtain the pill or condoms, but then not use the method.

To operationalise the concept of displacement, the analysis draws on detailed individual-level questions in the 2018 TDHS on Syrian women’s migration history. These include arrival year in Türkiye, number of migrations, Turkish language skills, temporary protection status, possession of an identity card, place of birth (country and governorate), and type of place of residence (camp/urban/rural). These indicators align with technical recommendations endorsed by the UN Statistical Commission for identification of refugees in national surveys.^104^

The table in Appendix 1 maps the framework’s seven dimensions of access against the available indicators in the 2018 TDHS on displacement and reasons for non-use of contraception. This helps to show which aspects of access can – and cannot – be analysed using the survey data. The indicators used to operationalise dimensions of access are not necessarily clear cut. For example, arrival year in the host country could interact with cognitive, administrative, and psychosocial accessibility.

Individual characteristics can also influence different dimensions of access in displacement. I therefore include a range of sociodemographic variables that are available in the data and have been shown to be associated with contraceptive use in conflict-affected settings.^37,105^ These are geographic region in the host country (Türkiye), sex of the household head, age group, educational attainment, and employment. I also consider Syrian women’s births in the last five years.

### Analytical strategy

The analysis is sequential and uses the Stata17 statistical software package.^106^ First, I use descriptive statistics to describe the two populations: married Syrian women in 2006 and married Syrian women in Türkiye in 2018. Then I analyse contraceptive use among Syrian women in Türkiye based on displacement dimensions of access. I use two sample tests of proportion (prtest in Stata)^106^ to analyse differences in overall use and method mix among displaced women compared to the population in the place of origin, a key step towards understanding potential mechanisms of access. For analysis at the governorate level, I use the governorate of residence for the 2006 Syria MICS and the (Syrian) governorate of birth for women in the 2018 TDHS.

The second phase of my analysis focuses on the 2018 TDHS and displaced Syrian women in Türkiye. I analyse patterns of contraceptive use and reasons for not using a contraceptive method, including women who have discontinued a method, and how this varies across dimensions of access and displacement experiences outlined in the framework. For example, can we use the data to understand whether cognitive accessibility is lower among women who cannot read and write in Turkish, or among those who have experienced multiple displacements? Do reasons for non-use vary across place of birth for Syrians in Türkiye? Finally, I look at current contraceptive users who state they would prefer to use an alternative method and why. In the discussion, I explain the results using the proposed framework of contraceptive access in displacement.

Within the constraints of the data, I make several analytical decisions. First, I analyse contraceptive use and access among currently married/in union women, rather than all women, as the 2006 MICS only asks these questions of married women. Second, I include only women with Syrian citizenship who were born in Syria from the 2018 TDHS sample, excluding 48 women with other citizenship and/or who were born outside Syria. Third, I analyse the datasets for Syrian women in Syria and Türkiye separately, meaning I can use the available survey weights to provide representative population-level statistics.

## Results

### Demographics of married women in the 2006 MICS and 2018 TDHS Syrian samples

Table 1 summarises the demographics of currently married or in union women in the 2006 MICS and 2018 TDHS Syrian samples.

**Table 1.** Sociodemographic characteristics of currently married/in union Syrian women aged 15–49 years in 2006 MICS and 2018 TDHS

	2018 TDHS	2006 MICS
	Weighted %	Weighted %
	*n* =1736	*n* = 13,619
Citizenship		
Syria	100	100
Country of birth		
Syria	100	No data
Arrival year in Türkiye		
2010 or before	0.1	N/a
2011	1.9	
2012	10.8	
2013	24.7	
2014	21.7	
2015	17.9	
2016	11.9	
2017	7.1	
2018	3.8	
Number of migrations		
1	0.8	No data
2	66.5	
3	21.9	
4+	10.8	
Temporary protection status issued^a^		
Yes	94.5	N/a
No	5.5	
Identity card issued^a^		
Yes	95.0	N/a
No	5.0	
Region (Türkiye)		
West	22.1	N/a
South	36.4	
Central	7.3	
East	34.2	
Residence^a^		
Urban	N/a	55.8
Rural		44.2
Non-camp	96.2	N/a
Camp	3.8	
Age group		
15-19	12.6	4.0
20-24	23.4	13.3
25-29	20.7	18.6
30-34	16.0	18.6
35-39	12.4	19.4
40-44	8.7	15.7
45-49	6.2	10.4
Education		
No education	13.3	19.5
Incomplete primary	5.8	N/a
Complete primary	34.9	
Incomplete secondary	17.9	
Complete secondary	21.9	
Higher	6.2	
Primary	N/a	34.8
Preparatory		24.1
Secondary		11.5
Academy		6.5
Higher		3.6
Turkish language (read and write)		
Yes	13.0	No data
No	87.0	
Currently employed		
No	93.7	No data
Yes	6.3	
Births in last 5 years		
0	33.1	No data
1	33.2	
2	27.6	
3	5.8	
4	0.3	
Total children ever born		
0	10.4	7.8
1	16.2	9.5
2	18.7	15.9
3	16.0	16.4
4	12.5	14.6
5	9.5	11.5
6	6.8	7.9
7+	9.9	16.4
Previous pregnancy outcomes (ever had)		
Miscarriage	27.8	No data
Abortion	5.0	
Stillbirth	2.8	

Notes: ^a^All respondents who resided in camps had been issued an identity card and temporary protection status. The 81 respondents who did not have an identity card, and 89 respondents who had not been issued temporary protection status, resided in urban settings. Of the 81 respondents who had not been issued an identity card, only one had been issued temporary protection status. Similarly, of the 89 respondents who had not been issued temporary protection status, only 9 had been issued an identity card.

The 2018 TDHS Syrian sample includes 2168 women aged 15–49 years old who were born in Syria and have Syrian citizenship, of whom around three-quarters were currently married (*n* = 1736). More than 99% arrived in Türkiye from 2011 onwards. 95% were issued temporary protection status in Türkiye and 4% lived in camps. In terms of geographic areas, the largest proportion was in the South of Türkiye, followed by the East and West, with the lowest proportion in Central Türkiye. The majority of women (66%) experienced two migration movements since age 12, with around one-third experiencing three or more. The majority of households were male-headed (93%). A minority of women was currently employed (6%) or could read and write in Turkish (13%). Around two-thirds of women (67%) had one or more births in the last five years, while one-third were nulliparous.

The 2006 Syria MICS sample includes 25,026 women aged 15–49 years old. The overwhelming majority of households were male-headed (92%). 54% of women were currently married or in union (*n* = 13,619). Of these, over half lived in urban areas (56%) and the governorate with the largest proportion of the population was Aleppo (23%).

### Contraceptive use among displaced Syrian women in 2018 vs. Syrian women in 2006

At the population level, current use of any contraceptive method was lower among displaced Syrian women in Türkiye in 2018 (43%), compared to Syrian women in 2006 (58%) (see Table 2). The use of modern contraceptives was lower (24% in 2018 vs 47% in 2006), while the use of traditional methods was higher (19% in 2018 vs 12% in 2006). In Table 2, 95% confidence intervals for the percentage point difference in contraceptive use between 2006 and 2018 are only provided at the total level, rather than by governorate, due to the small sample size of married women for some governorates in the 2018 TDHS.

**Table 2.** Contraceptive use (modern/traditional/any method) by Syrian governorate in 2006 and 2018

Contraceptive use (%)														
	Modern				Traditional				Any				Total number married women	
Total	2006 MICS	2018 TDHS	*95%* CI^a^	*P*	2006 MICS	2018 TDHS	*95% CI*^a^	*p*	2006 MICS	2018 TDHS	*95% CI*^a^	*p*	2006 MICS	2018 TDHS
	46.6	24.1	[−24.7, −20.3]	<0.001	11.7	19.0	[5.4, 9.2]	<0.001	58.3	43.1	[−17.7, −12.7]	<0.001	13,619	1736
Governorate														
Daraa	38.0	21.1			5	31.6			43.1	52.6			713	7
Deir Ezzor	35.7	14.1			2.1	15.4			37.8	29.5			614	82
Aleppo	53.3	25.6			6.5	17.7			59.8	43.3			3148	997
Hama	41.7	23.9			14.5	22.2			56.2	46.1			964	112
Hassake	26.7	30.1			17.3	16.4			44.1	46.4			733	50
Homs	46.8	22.5			12.6	24.9			59.5	47.4			1209	87
Idleb	45.1	26.3			12.1	20.1			57.2	46.4			811	171
Quneitra	33.3	0.0			3.7	100			37.0	100			81	1
Lattakia	48.2	15.2			22.3	30.4			70.5	45.7			685	70
Raqqa	28.3	14.4			7.9	15.0			33.7	29.4			523	63
Rural Dam	47.0	20.6			17.4	35.3			64.4	55.9			2071	12
Sweida	51.2	0.0			23.7	0.0			74.9	0.0			279	1
Damascus	60.8	25.6			10.1	21.0			70.8	46.6			1181	80
Tartous	44.0	0.0			21.9	0.0			65.9	0.0			607	3

Notes: Governorate in 2018 TDHS refers to place of birth in Syria. Governorate in 2006 Syria MICS refers to current residence. Modern methods of contraception: female sterilisation, pill, IUD, injectables, male condom, diaphragm / foam / jelly, LAM. Traditional methods of contraception: periodic abstinence / rhythm, withdrawal.

^a^ (% point difference in rate).

In terms of method type, use of both short- and long-acting methods of modern contraception was lower among the displaced population compared to Syrian women in 2006 (see Table 3). Across this period, IUD (26%–13%) and pill (13%–6%) use show a marked decline among the displaced population, while use of withdrawal (2%–18%) and no reported method (42%–57%) was higher. The number of women in the 2018 TDHS reporting other methods such as female sterilisation, injectables, diaphragm, and periodic abstinence were small so results should be interpreted with caution.

**Table 3.** Contraceptive use by method type in 2006 and 2018

Contraceptive method	2006 MICS *n* = 13,619 %	2018 TDHS *n* = 1736 %	95% CI *(% point difference in rate)*	*p*
Total	100	100	–	-
Sterilisation (female)	1.2	2.0	[0.1, 1.5]	0.005
Pill	12.9	6.3	[−7.9, −5.3]	<0.001
IUD	25.7	13.0	[−14.4, −11.0]	<0.001
Injectables	0.9	0.4	[−0.8, −0.2]	0.032
Condom (male)	1.6	2.3	[0.0, 1.4]	0.033
Diaphragm/foam/jelly	0.2	0.1	[−0.3, 0.1]	0.366
LAM	4.0	–	–	-
Periodic abstinence / rhythm	9.2	0.6	[−9.2, −8.0]	<0.001
Withdrawal	1.7	18.3	[14.8, 18.4]	<0.001
Other	0.8	–	–	-
No method	41.7	56.9	[12.7, 17.7]	<0.001

At the governorate level, the largest sample size in the 2018 TDHS is for women who were born in Aleppo (*n* = 997), consistent with geographic proximity and displacement patterns to Türkiye. Table 2 shows that modern contraceptive use among women in 2018 who were born in Aleppo (26%) was half that of women who lived in Aleppo in 2006 (53%).

### Variation in contraceptive use among displaced Syrian women in 2018: a lens on access

Table 4 shows the variation in contraceptive use (modern/traditional/any method) among married Syrian women displaced to Türkiye by sociodemographic characteristics. As an indicator of administrative access, modern contraceptive use was higher among women who had an identity card issued (25%) compared to those who did not (12%). In terms of residence type (and geographic access), the use of modern contraceptives was relatively similar among those who lived in a camp (23%) versus non-camp locations (24%), while the use of traditional methods was higher among women in camps (26% vs 19%). The use of traditional (vs modern) methods was also higher for married adolescent and young women aged 15–24, whereas from ages 25 onwards, the use of modern methods was higher.

**Table 4.** Contraceptive use (modern / traditional / any method) among married Syrian women in Türkiye in 2018, by sociodemographic characteristics

Total		All %	Modern %	Traditional %	Total number married women
		43.1	24.1	19.0	1736
Arrival year in Türkiye	2010 or before	50.0	50.0	0	2
	2011	45.1	32.3	12.9	47
	2012	43.0	22.5	20.5	217
	2013	47.3	27.2	20.1	439
	2014	48.8	30.2	18.6	359
	2015	41.6	22.2	19.4	302
	2016	39.8	20.5	19.3	194
	2017	32.5	14.0	18.4	114
	2018	19.3	8.1	11.3	62
Number of migrations	1	7.7	7.7	0.0	13
	2	42.4	24.1	18.4	1142
	3	44.7	24.5	20.2	399
	4+	46.5	25.1	21.5	182
Temporary protection status issued	Yes	43.7	24.8	18.9	1647
	No	32.6	13.5	19.1	89
Identity card issued	Yes	43.7	24.8	19.0	1655
	No	30.9	12.4	18.5	81
Sex of household head	Male	44.0	24.7	19.3	1621
	Female	30.3	16.2	14.1	115
Region (Türkiye)	West	46.1	23.1	23.1	356
	South	43.0	23.2	19.8	638
	Central	38.4	20.5	18.0	117
	East	42.2	26.6	15.6	625
Residence	Non-camp	42.8	24.2	18.7	1550
	Camp	48.9	22.6	26.3	186
Age group	15–19	20.9	8.9	12.0	209
	20–24	33.5	15.0	18.5	399
	25–29	46.9	25.8	21.0	357
	30–34	51.7	33.7	18.0	282
	35–39	57.6	34.8	22.8	225
	40–44	57.1	34.8	22.3	154
	45–49	40.4	22.5	17.9	110
Education	No education	37.3	21.7	15.5	230
	Incomplete primary	45.3	31.1	14.2	97
	Complete primary	42.7	25.1	17.6	614
	Incomplete secondary	42.7	23.7	19.0	308
	Complete secondary	46.7	25.0	21.7	381
	Higher	44.0	15.4	28.6	106
Turkish language (read and write)	Yes	49.5	26.3	23.2	228
	No	42.1	23.8	18.3	1508
Currently employed	Yes	39.2	21.7	17.4	108
	No	43.3	24.3	19.1	1628
Births in last 5 years	0	37.4	22.5	14.9	570
	1	45.8	24.4	21.4	571
	2	47.7	26.5	21.2	484
	3	40.8	22.6	18.3	105
	4	0.0	0.0	0.0	6

### Reasons for non-use: constraints on contraceptive access in displacement?

Table 5 presents the main reasons for non-use by different dimensions of access among married Syrian women displaced to Türkiye: (1) women currently using a method of contraception who would prefer to use a different method, (2) non-users of contraception, and (3) women who discontinued a contraceptive method in the five years preceding the survey (including both current users and non-users).

**Table 5.** Main reason for not using a contraceptive method among married Syrian women in Türkiye in 2018, by dimension of access

Dimension of access	Reasons	Users (not using preferred method) %	Non-users (not using any method) %	Last discontinuation (users and non-users) %
		*n* = 89	*n* = 684	*n* = 562
Availability	Preferred method not available	–	–	–
	No method available	–	–	–
Affordability	Costs too much	2.4	0.3	0.4
Cognitive accessibility	Knows no method	–	0.5	–
	Knows no source	4.0	0.2	–
	Does not know how to use	1.2	–	–
	Interferes with body	–	–	–
	Inconvenient to use	–	–	–
	Fears side effects	19.3	1.5	20.9
	Health concerns	44.2	4.7	5.2
Psychosocial accessibility	Respondent opposed	–	–	–
	Husband/partner opposed	6.0	2.3	2.4
	Others opposed	–	–	–
	Religious prohibition	–	0.9	–
	Up to God/fatalistic	–	0.7	0.2
Geographic accessibility	Lack of access/too far	–	–	0.8
Administrative accessibility	n/a	–	–	–
Perceived quality of care	Interferes with body	–	–	–
	Inconvenient to use	–	–	1.0
	Side effects	–	–	–
	Health concerns	–	–	–
	Wanted more effective method	–	–	3.6
	Doctor did not advise	5.6	–	–
	Preferred method not available	–	–	–
	No method available	–	–	–

Other	Other	17.3	9.7	3.4
Fertility-related reasons	Pregnant	–	–	16.6
	Postpartum, breastfeeding	–	24.6	–
	Wants more children	–	29.4	42.4
	Menopausal, hysterecotomy	–	3.1	1.0
	Infrequent sex	–	5.6	2.2
	Not having sex	–	4.5	–
	Subfecund, infecund	–	12.0	–

12% of married women who were currently using a modern or traditional method of contraception reported that they would prefer to use a different method. This proportion was higher among women using a traditional method (16%) compared to those using a modern method (9%). The most popular alternative preferred method was the IUD (53%), followed by the pill (17%). The main reasons for not currently using the alternative method were due to cognitive accessibility and perceived quality of care, as well as psychosocial accessibility; health concerns (44%), side effects (19%), husband opposition (6%), or because the doctor did not advise it (6%).

More than half (57%) of married Syrian women in Türkiye reported they were not currently using a contraceptive method. Available data for non-users shows that most women expressed fertility-related reasons for not using a method. For example, because they were postpartum or breastfeeding (25%), wanted more children (29%), were having infrequent sex or not having sex (10%), or perceived themselves (or their husband) as sub/infecund (12%), i.e. they did not consider themselves to have a need for contraception. Other reasons for not using a contraceptive method that fit into different dimensions of the contraceptive access framework, were less commonly reported than fertility-related reasons. Different components of cognitive accessibility, particularly health concerns and fear of side effects, were notable reasons. However, less than 2% of women reported barriers such as religion, lack of knowledge on method or source, and cost. No observations were available for other response options, including geographic accessibility. It is unclear whether the reasons were not reported (or the interviewer did not code responses to these options), or whether responses were aggregated into the substantial “other” category (10%). It is also important to note that the 2018 TDHS only captures the main reason for not using contraception, when likely there may be multiple overlapping reasons.

The main reasons for discontinuing a contraceptive method in the previous five years were also mainly due to fertility-related reasons. 42% of women wanted to become pregnant, while 17% became pregnant due to method failure (primarily withdrawal). Certain dimensions of access, specifically side effects and health concerns (i.e. cognitive accessibility and perceived quality of care), were the main reasons for one quarter of women who discontinued a method.

## Discussion

This paper operationalises the concept of contraceptive access in the context of displacement, presenting a conceptual framework and empirical case study. I revisit references to “access” in existing SRH frameworks for conflict and displacement settings^77,78^ and offer a structured approach for researchers and practitioners to better understand different aspects of contraceptive access in displacement settings (see Figure 1). I optimise nationally representative survey data to test components of the framework using the case study of Syrians in Türkiye, analysing how reasons for contraceptive non-use relate to different aspects of access. I also show how patterns of contraceptive use among displaced Syrians in Türkiye differ from the most closely comparable non-displaced population. This paper advances both theoretical and substantive understandings of contraceptive access in displacement, as well as methodological implications for measurement, each of which I discuss here.

### Connecting the framework and the case study of Syrians in Türkiye

The case study of Syrians in Türkiye provides an opportunity to test which of the framework’s seven dimensions of access are most relevant in this displacement setting. For women who were using contraception in 2018, but not their preferred method, the key constraints were mainly due to cognitive accessibility and perceived quality of care, specifically concerns about side effects and other health concerns. The discrepancy between preferences and realising that preference suggests limits to contraceptive autonomy in displacement, as evidenced in other settings.^107^ This finding also challenges the notion that current users of contraception do not face barriers to access, as well as highlighting the potential role of underlying structural and systemic issues that may constrain different dimensions of access. Fear of side effects and other health concerns (i.e. cognitive accessibility and perceived quality of care) were also reasons given by Syrian women in 2018 who had discontinued a method within the last five years or who did not currently use contraception. However, the main reasons for non-use among this group were overwhelmingly fertility-related and not due to access.

Comparisons between use of modern and traditional methods of contraception offer further insights into which dimensions of the framework may be important in this setting. Overall, the displaced population in 2018 reported lower use of modern contraceptives and higher use of traditional methods, compared to the population in the place of origin in 2006. While women may be relying on – or are exercising a preference for – traditional contraceptive methods in displacement, other factors may be at play. Among the displaced population in 2018, the use of modern methods was higher among those with an identity card, potentially suggesting the importance of administrative accessibility to modern methods of contraception. Furthermore, the use of traditional methods was comparatively higher among women who lived in camps; this may be explained by constraints to the availability of services and/or geographic accessibility. Age was also a distinguishing factor in method type, with a higher use of traditional methods compared to modern methods among displaced adolescents and youth in Türkiye in 2018. This finding about age may point to a lack of inclusive services, specifically adolescent- and youth-friendly services, reflecting potential constraints across multiple dimensions of the framework (psychosocial accessibility, perceived quality of care, availability, and administrative access). This is consistent with research that documents insufficiencies in adolescent- and youth-friendly SRH services for Syrian refugees in other countries, as well as young people globally.^108–110^

The case study illustrates the added value of the framework, specifically a structured approach for researchers and practitioners to better understand different aspects of contraceptive access in displacement settings. A displacement lens offers wider transferability of the framework to diverse geopolitical settings compared to other framings. For example, integration is often used when researching EU cities, however, it would not be appropriate for all displacement contexts. While this case study relates to people who have fled conflict, the framework could also be applied to acute displacement in more active conflict settings, including internal displacement.

It is useful to reflect on how the framing of displacement, especially in protracted settings such as Türkiye, affects how we think of the agency of and integration possibilities for those displaced. I want to unambiguously emphasise that displaced people show extraordinary resilience and agency, and a range of durable solutions can be available.^18^ The continuum of needs and agency over time supports the rationale to delink displacement from a humanitarian, life-saving lens. In line with the integration literature, the framework incorporates and emphasises multidimensional elements of host society opportunity structures (such as social security coverage) in shaping displaced people’s outcomes, not only personal characteristics.^101^

The framework does not aim to be exhaustive, nor can it provide a complete picture. This is partly due to the current data landscape and methodological constraints discussed below. However, data on displaced populations is rapidly evolving, thanks to efforts by the Expert Group on Refugee, IDP and Statelessness Statistics (EGRISS), Joint Data Centre on Forced Displacement (JDC), and national statistics offices, among others. As new higher quality datasets emerge, the framework can be applied to a range of displacement settings and tested and iterated. For example, the framework could be used as part of a comparative study to explore which aspects of access are more relevant in internal displacement compared to refugee settings. The framework could also help to identify specific challenges to SRH service access among displaced adolescents and youths, in a comparative study with the host population.

The study’s findings are consistent with literature on Syrians in neighbouring host countries. This includes different SRH outcomes of Syrians in Lebanon pre- and post-displacement,^111^ as well as the role of health concerns in contraceptive non-use among Syrian refugees in Jordan in 2017–2018.^112^ A mixed methods study of Syrian refugee youth in Jordan between 2022 and 2023 found that recent use of modern contraceptives was higher among those living in a host community, compared to Syrian refugee youth living in refugee camps.^113^ This disparity was explained by more salient social norms and limited knowledge about reproductive health among refugee youth in camp settings, together with greater availability of public and private health providers as well as pharmacies in host communities.^113^ Interestingly, the study found that recent use of any contraceptive method, as well as the withdrawal method, was higher among Syrian refugee youth living in a host community compared to refugee camps – this may indicate a difference between women aged 15–49 versus youth specifically.

The results align with evidence from other conflict settings beyond the Syrian crisis. For example, using DHS data from Colombia, Svallfors and Bingley^114^ found a negative relationship between modern contraceptive use and conflict intensity.^114^ This was only partially because of increased fertility demand, with the authors citing reduced access to contraceptive commodities and services as a potential explanatory mechanism.

### Methodological takeaways: strengthening understandings of contraceptive access with high quality data

Applying the framework to the case study of Syrians displaced to Türkiye helps to illustrate several methodological considerations for measurement. The analysis shows the value of including individual-level questions on migration history in national surveys, such as those in the 2018 TDHS, and the powerful information they offer about displacement timing, duration, and setting (i.e. geographic and administrative accessibility), and how this relates to health outcomes. Whilst researchers must optimise the available data, inadequate SRH data in displacement settings continues to limit understandings.^36,37^

For example, evidence from low- and middle-income countries more generally affirms that concerns regarding side effects or health risks are some of the main barriers to contraceptive access, regardless of displacement status.^115^ This may mean that survey questions are not picking up the nuances of barriers faced by displaced people for several reasons. First, the TDHS data captures only the main reasons for non-use, rather than the complex realities of contraceptive decision making. This means that secondary or multiple reasons that influence contraceptive decision making are not captured and are instead reported as zero in the data. The “other” response option was a substantial proportion in reasons for non-use, which may potentially mask various dimensions of access. Furthermore, data to measure different aspects of access in the framework, particularly variables on quality of care, have not been routinely collected in the past. For example, questions on side effects and counselling on other contraceptive methods (see, e.g., questions 318, 321 and 323 in the DHS Model Questionnaire Phase 7) were not asked as part of the 2018 TDHS. While it is important to minimise the length of questionnaires to reduce the burden on participants, the inclusion of questions on quality of care links to wider debates around contraceptive measurement with an autonomy- and rights-based lens.^107,116,117^ Incorporating questions on quality of counselling, as well as allowing survey respondents to report more than one reason for contraceptive non-use, could help build a more comprehensive picture.

There are also many ways to capture and categorise the complex realities of access. Senderowicz and Maloney’s pioneering work on unmet need reflects this, outlining three alternative definitions of supply-side and demand-side unmet need (strict, moderate, broad).^118^ The proposed framework for displacement in this paper takes a broader approach, acknowledging that demand for services can overlap with some dimensions of access, particularly psychosocial accessibility and perceived quality of care.

An important part of optimising existing data is recognising its limitations. First, the two populations are not the same set of women, meaning there may be underlying differences in those who are displaced. I am unable to account for any changes in secular trends in contraception and method mix since 2006, as there is limited other data on contraceptive preferences and patterns for Syrians throughout the conflict. Secondly, the MICS3 (used in the 2006 Syria MICS) and 2018 TDHS survey tools are not identical. For example, the 2006 Syria MICS does not include questions on reasons for contraceptive non-use. This means I am unable to analyse how dimensions of contraceptive access differ between the two populations. Additionally, the 2006 Syria MICS only asks questions about contraceptive use to currently married/in union women, rather than all women. Third, to optimise the available data, I must use proxies for some variables. For example, place of birth for displaced Syrian women is not synonymous with place of origin among the non-displaced population (for sub-national analyses at the governorate level). Finally, while the findings are based on nationally representative survey data, the pattern of displacement from Syria to Türkiye is not the same for all governorates. Syrians were also significantly displaced to two neighbouring countries, Jordan and Lebanon, depending on geographic proximity and social networks. Ideally, I would include Syrians in Jordan and Lebanon in the study’s analyses, but this is limited by data availability; the 2017/18 Jordan DHS includes Syrians, but not a question on place of birth, and the 2023 Lebanon MICS data are currently unavailable.

### Areas for future research

The study’s findings highlight several areas for further investigation. First, the proportion of women in the 2018 TDHS with certain displacement characteristics, such as those who are not under temporary protection status or those living in camp settings, is relatively small. It would therefore be informative to study these dimensions of administrative and geographic accessibility in other countries, such as where the proportion of camp-based displaced persons is higher. Second, these analyses rely fully on survey data, which means data on some elements of the conceptual framework are missing. In future analyses, multi-level modelling could be used to incorporate data on the existence and content of relevant laws and policies, restrictions around access to care, and various health system factors, to better capture different aspects of the conceptual framework in a range of settings. Third, qualitative research – either stand-alone or part of mixed methods study designs – could help to offer insights on areas of the framework that were identified as important, such as cognitive accessibility and perceived quality of care, as well as aspects of the framework for which survey data are not available. Qualitative or participatory approaches could also be valuable in exploring potential preferences for traditional (or other) contraceptive methods post-displacement. Finally, the 2018 TDHS only provides quantitative evidence of contraceptive use among women, so it would be interesting to explore this among other gender identities including men. The framework offers a structured approach to explore which aspects of access may be gendered or especially pertinent to men.

## Conclusion

This paper advances theoretical and substantive understandings of contraceptive access in displacement settings, as well as methodological implications for measurement. The specific lens on displacement offers a narrower and more constructive focus than humanitarian or fragile settings more broadly and recognises how displacement transcends the simplistic humanitarian-development dichotomy.

I make three main contributions to the literature. First, I offer a framework that can be used and iterated by other researchers to better understand different aspects of contraceptive access in displacement settings. Second, I use the case study of Syrians in Türkiye to test the framework by optimising nationally representative survey data. Finally, I offer a critical reflection on what is being measured. Together, I seek to make a wider contribution towards improving access to comprehensive contraception in displacement for those who wish to use it, in an effort to help realise rights and save lives.

The results show that in this context, the most relevant dimensions of contraceptive access in the framework were cognitive accessibility and perceived quality of care, specifically fear of side effects and other health concerns. These factors, underlined by wider structural and systemic issues, prevent displaced Syrian women from accessing their preferred methods of contraception, even among current users. The study’s findings help to address evidence gaps on SRH in displacement, specifically analysis of contraceptive use among a displaced population, compared to the most closely comparable non-displaced population.

The case study also offers broader takeaways about data and evidence, notably that understandings of contraceptive access in displacement will continue to be partial with the currently available metrics. The 2018 TDHS illustrates the value of incorporating questions on place of birth and place of origin as standard for displacement samples. To provide a comprehensive picture, surveys could capture more than one reason for contraceptive non-use, use cognitive interviews to strengthen coding of the “other” category for reasons for non-use, and incorporate questions on quality of contraceptive counselling.

## Supplementary Material

Supplemental material

## Data Availability

MICS data referenced in the paper are available from UNICEF at https://mics.unicef.org/surveys, with free registration. TDHS data is available to researchers through a data request to the Haceteppe University Institute of Population Studies at https://tnsaveri_tdhsdata.hacettepe.edu.tr/request.php.

## Provenance statement

This article was not commissioned and went through external peer review.
