# Supplementary material for: Contraceptive access in displacement settings: a quantitative study of Syrians displaced to Türkiye
Source: Sex Reprod Health Matters. 2026 Jan 7;33(1):2607838. doi: 10.1080/26410397.2025.2607838 (PMC12943824; doi:10.1080/26410397.2025.2607838)
Supplement: Supplemental material [file ZRHM_A_2607838_SM1635.docx]

## Additional file: Appendix 1

This appendix provides more details on the methodology to develop the framework outlined in Figure 1, as well as a table with examples from the literature for further explanation.

The methodology to develop the framework was sequential. First, I ran a search for scoping and systematic review articles that included literature on contraceptive access in conflict displacement settings. The search strategy used key words (contraception, family planning, sexual and reproductive health, access, displacement, refugee, internal displacement, asylum seeker, stateless, systematic, scoping, review), as well as their synonyms, abbreviations, and spelling variations. The search was run in PubMed and Google Scholar databases. To minimise risk of omissions, I cross-checked the search results against a database of literature sourced through daily Google Scholar alerts on “sexual and reproductive health” and “forced displacement” since October 2020. Following the search, I screened abstracts and titles based on the following inclusion criteria: 1) scoping or systematic review; 2) English language publications; 3) published in peer-reviewed journal between 1/1/1990 and 31/12/2023; 4) includes studies that provide quantitative and/or qualitative evidence on contraceptive access among refugees, IDPs, asylum seekers, undocumented migrants, stateless and other categories of displaced people. The screening resulted in the inclusion of 21 systematic and scoping reviews (Amiri et al., 2020; Amodu et al., 2020; Casey, 2015; Çöl et al., 2020; Davidson et al., 2022; Desrosiers et al., 2020; Donnelly et al., 2023; Egli-Gany et al., 2021; Ekezie et al., 2020; Fatemi & Moslehi, 2021; Hossain & Dawson, 2022; Ireland et al., 2021; Ivanova et al., 2018; Jennings et al., 2019; Larrea-Schiavon et al., 2022; Munyuzangabo et al., 2020; Ojeleke et al., 2022; Sawadogo et al., 2023; Singh et al., 2018; Soeiro et al., 2023; Warren et al., 2015). Next, I extracted data from each review paper, including relevant factors from studies in the reviews that were found to facilitate or constrain access to contraception, structured against Sochas’ seven dimensions of access: cognitive, psychosocial, geographic, and administrative accessibility; affordability; perceived quality of care; and availability (Sochas, 2020). Finally, I synthesised the data in a framework (see table below) to offer a structured approach to understanding aspects of contraceptive access in conflict displacement settings in relation to seven key dimensions of geographic and social access. The table includes illustrative examples, selected from studies identified in the reviews.

Table: Aspects of contraceptive access in conflict displacement settings and associated DHS measures

| Dimension of access | Definition | SRH access in displacement | | | | DHS data / variable | |
| --- | --- | --- | --- | --- | --- | --- | --- |
|  |  | Constrain or facilitate | Constrain | Facilitate | Example(s) from literature | Displacement characteristics | Reasons for not using contraception |
| Availability | ‘The relationship of the volume and type of existing services to the clients’ volume and types of needs’^1^ | - Comprehensive method choice - Sustained and timely funding for services - Hours/days of service operation e.g. not clashing with other food or financial distributions - Referrals between community and different health system levels | - Infrastructure damage - Inadequate service coverage and narrow focus on specific services and populations - Supply chain disruption for commodities - Loss of skilled health workers | - Proliferation of humanitarian actors and services - Service provision by trained displaced health workers helps continuity of services | In Türkiye, the Ministry of Health and UNFPA established dedicated refugee health and women’s centres for Syrians (Çöl et al., 2020). |  | Preferred method not available  No method available |
| Affordability | ‘The relationship of prices of services to the clients’ income, ability to pay and health insurance’^1^ | - Employment status of displaced people - Financial resources | - Cost of services higher for non-nationals - Out-of-pocket expenses e.g. travel costs | - Social security or health insurance coverage - Free or subsidised services | The cost of contraceptive commodities was a barrier experienced by refugee youths living in a camp in Nigeria, after the camp clinic and free services were discontinued (Okanlawon et al., 2010), cited by (Ivanova et al., 2018). |  | Cost |
| Cognitive accessibility | ‘Extent to which potential clients are aware of the locations of service (…) points and of the services available at these locations’^2^ | - Knowledge of available services - Language and access to interpreters - Phone network and charging facilities - Range and suitability of information channels about health services - Community outreach, peer educators, and school-based activities for young people - Health literacy, including misinformation about side effects of methods - Social networks |  |  | Refugees faced language barriers with contraceptive providers in Eastleigh, Kenya and Kuala Lumpur, Malaysia (Tanabe et al., 2017), cited by (Davidson et al., 2022). | Turkish language skills (read and write)  Arrival year in Türkiye* | Knows no method  Knows no source  Does not know how to use  Interferes with body  Inconvenient to use  Side effects  Health concerns |
| Psychosocial accessibility | ‘Extent to which clients are constrained by psychological, attitudinal or social factors in seeking out (…) services’^2^ | - Social, cultural and religious norms in place of origin and arrival e.g. pronatalist attitudes - Management of security risks - Participation of local initiatives and civil society in the co-design and evaluation of service delivery - Decision making autonomy - Social capital and support | - Risk (perceived or real) of detention or deportation by control services due to legal status - Stigma and discrimination related to displacement status, gender, sexual orientation, and pregnancy outside of marriage | - Integration with non-SRH services, increasing privacy when seeking care - Confidentiality e.g. anonymised electronic record keeping, as records can get lost during violent attacks | Attacks on health facilities in IDP sites in Burundi and northern Uganda (Chi et al., 2015), cited by (Amodu et al., 2020). |  | Respondent opposed  Husband/partner opposed  Others opposed  Religious prohibition  Up to God/fatalistic |
| Geographic accessibility | ‘The relationship between the location of supply and the location of clients, taking into account client transportation resources and travel time, distance and cost’^1^ | - Residence type (camp/ urban areas/ informal settlements/ detention centres) - Distance to services and living in (under)served areas | - Frequent and involuntary mobility limits continuity of care and follow up - Curfews or other movement restrictions (e.g. check points) | - Community based programmes and mobile clinics - Damage to roads and other transport infrastructure | Instability and associated population movements limits services requiring long-term follow-up (Casey, 2015). | Place of residence (camp/urban/rural)  Number of migrations | Lack of access/too far |
| Administrative accessibility | ‘The relationship between the manner in which the supply resources are organised to accept clients and the clients’ ability to accommodate to these factors, and the clients’ perception of their appropriateness’^1^ | - Laws and policies in place of arrival - Legal status and entitlements, including eligibility to receive services e.g. exclusion of undocumented migrants - Possession of civil registration documents and requirement for official documentation to access services - Health centre policies e.g. on chaperones - Involvement of accountable authorities, including local government - Age |  | - Marriage | Introduction of security cards prevented Syrians' access to reproductive health clinics in Jordan (Juraibei, 2016), cited by (Amiri et al., 2020). | Temporary protection status  Identity card issued |  |
| Perceived quality of care | ‘Clients’ perception of the extent to which they are likely to receive effective care once they access a facility’^3^ | - Type of service provider e.g. public, private, NGO - Inclusive services e.g. age-appropriate - Confidentiality of services - Profile of health workers e.g. gender, age, culturally competent, from affected population - Competence of staff - Condition of facilities - Wait times |  |  | Refugees with disabilities in Kenya and Uganda experienced negative and disrespectful provider attitudes (Tanabe et al., 2015), cited by (Ojeleke et al., 2022). |  | Interferes with body  Inconvenient to use  Side effects  Health concerns  Wanted more effective method  Doctor did not advise  Preferred method not available  No method available |

Notes:

1 (Penchansky and Thomas, 1981)

2 (Bertrand et al., 1995)

3 (Sochas, 2020)

* All variables are taken from the 2018 TDHS woman’s questionnaire, except ‘arrival year in Türkiye’ from the household questionnaire
